# Editorial: Hydrothermal microbial ecosystems

**DOI:** 10.3389/fmicb.2015.00884

**Published:** 2015-09-01

**Authors:** Andreas Teske, Anna-Louise Reysenbach

**Affiliations:** ^1^Department of Marine Sciences, University of North CarolinaChapel Hill, NC, USA; ^2^Biology Department, Portland State UniversityPortland, OR, USA

**Keywords:** hydrothermal vent, extremophiles, biogeography, chemosynthesis, archaea, bacteria, Guaymas basin

The papers in the “Hydrothermal Vent” research topic cover a range of microbiological research in deep and shallow hydrothermal environments, from high temperature “black smokers,” to diffuse flow habitats and episodically discharging subsurface fluids, to the hydrothermal plumes. Together they provide a snapshot of current research interests in a field that has evolved rapidly since the discovery of hydrothermal vents in 1977.

The topic opens with a review by Dick et al. (2013) on hydrothermal plumes and their microbial communities in the deep sea. The review synthesizes recent advances in the microbial ecology, physiology, and genomics of the mostly chemosynthetic bacteria that have adapted to the supply of carbon and energy sources in the turbulently mixed vent plume. These pelagic communities are distinct from those in hydrothermal chimneys, sediments and the subsurface, yet they show some taxonomic and physiological linkages. This review is followed by four papers that shed new light on microbial ecosystems in mixed vent fluids. The high-flow example is represented by sulfide-rich subsurface vent fluids discharging in “snow blower” bursts dominated by sulfur-oxidizing, microaerobic, and often moderately thermophilic Epsilonproteobacteria (Meyer et al., 2013a). Similar epsilonproteobacterial communities, dominated by the chemosynthetic, sulfur-oxidizing genera *Sulfurimonas* and *Sulfurovum*, were also found in cool, diffusive flow at the same location, Axial Seamount on the Juan de Fuca Ridge (Akerman et al., 2013). The authors proposed that both diffuse flow and episodic high-flow events tap into the same subsurface community of microaerobic sulfur oxidizers. Epsilonproteobacteria accounted again for most of the microbial taxa found in diffuse flow samples from the basalt-hosted vent sites of 9°N East Pacific Rise, but not in similar habitats in the organic-rich hydrothermal sediments of Guaymas Basin in the Gulf of California, where other bacterial groups and hyperthermophilic archaea predominated (Campbell et al., 2013). Changing flow paths and reservoirs for subseafloor mixed fluids and their microbiota can also change the composition of venting microbial communities over time as reported by Kato and colleagues. At Suiyo Seamount in the Izu-Bonin Arc within the Western Pacific, a hot and reducing subsurface reservoir accessed by drilling underwent increasing seawater in-mixing over several years. Consequently, the crustal fluid community of sulfur-oxidizing chemolithotrophs changed to a mixed assemblage harboring abundant marine heterotrophic bacteria (Kato et al., 2013).

While deep-sea hydrothermal vent sites are difficult to access and to sample, shallow water hydrothermal vents that can be reached by scuba divers provide an accessible alternative environment to study. Perhaps the best-investigated of these shallow-water vents are found on the coast of the Greek island Milos in the Aegean Sea, where hot, briny, reducing, CO_2_-rich vent fluids permeate the sandy seafloor sediments of Paleochori Bay. Giovannelli et al. (2013) show that in the surficial sediments the microbial community is dominated by Epsilonproteo-bacteria (genus *Sulfurovum*) similar to those seen in deep-sea vents; however proteobacterial lineages that are distinct from those of deep-sea vents were also detected. In contrast, in the deeper sediment layers of this sample vent field, Price et al. (2013) find Epsilonproteobacteria, but also Firmicutes, Planctomycetes, and Bacteroidetes coexisting with thermophilic archaea. In addition, arsenite-oxidizing bacteria were identified by functional gene analysis in these arsenite-rich sediments. Site-specific adaptations of vent communities are also the focus of Bayraktarov et al. (2013), as they examine the pH preferences of sulfate-reducing microbial communities in the Milos vent sediments. In the proximity of CO_2_-rich, low-pH fluids, the sulfate-reducing microbial communities show maximal activities at a distinctly lower pH range than at less acidic control sites not impacted by CO_2_-rich vent fluids.

Among deep-sea vent sites, the sediment-covered Guaymas Basin hydrothermal vents in the central Gulf of California is unusual; the buried organic matter in these sediments are hydrothermally processed to petroleum compounds, low-molecular weight organic acids, short-chain alkanes, methane and ammonia. These substrates sustain a unique microbial ecosystem that combines the characteristics of communities from hydrocarbon seeps and mid-ocean ridge hydrothermal vents. Meyer and colleagues demonstrate that the microbial populations in Guaymas sediments show a high degree of microbial connectivity and population overlap within an area of a few 100 m—a consequence of vigorous venting, rapid dispersal via bottom currents, and closely spaced hydrothermal features (Meyer et al., 2013b). Abundant aromatic and aliphatic hydrocarbons in Guaymas Basin sediments and chimneys enrich for microbial specialists—especially sulfate-reducing bacteria—that utilize hydrocarbons, remineralize them to CO_2_, or assimilate them into microbial biomass; these communities feature prominently in metagenomic analyses of hydrocarbon-rich Guaymas chimneys (He et al., 2013). The ammonia-rich and methane-rich hydrothermal fluids at Guaymas sustain ammonia-oxidizing bacteria (anammox) and methane- oxidizing archaea (ANME) in the surficial sediments. The anammox bacteria combine ammonia and nitrite to N2, and are usually better known from marine oxygen minimum zones and stratified water columns (Russ et al., 2013). A recently identified, high-temperature tolerant clade of the cosmopolitan seep methane- oxidizing archaeal lineage ANME-1 is widespread at Guaymas Basin. The “ANME_1Guaymas” have been enriched using *in-situ* colonization chambers that were placed into surficial hydrothermal sediments on the seafloor (Callac et al., 2013). One of the relatively few analogous environments to Guaymas Basin, the alkane-rich hydrothermal sediments of the Middle Valley vent field on the northern Juan de Fuca ridge, provide the opportunity to study similar microbial communities and processes. In anaerobic batch reaction incubations, short alkanes (ethane, propane, butane) were consumed within a mesophilic to thermophilic temperature range, probably by sulfate-reducing bacteria (Adams et al., 2013).

Hydrothermal vents are suitable model systems to explore microbial biogeography because they are island habitats in the deep sea; their thermophilic, chemosynthetic or special substrate-adapted microbial inhabitants likely cannot survive, and certainly cannot thrive in the cold, oxidized sediments and bottom waters of the deep sea that separate hydrothermal hot spots. In this volume several papers evaluate the controls of microbial community structure, geographic distance vs. environmental selection pressures in different habitats such as tubeworm trophosomes, sulfides, and ferric iron hydroxides. Sulfur-oxidizing endosymbionts of vestimentiferan tubeworms, which thrive in seep and vent habitats, showed a clear separation into different Gulf of Mexico and Mediterranean genotypes by 16S rRNA gene, mitochondrial cytochrome C oxidase, and functional genes of the two coexisting CO_2_ assimilation pathways that coexist in the endosymbiont, the Calvin-Benson-Bassham and the reverse TCA cycle (Thiel et al., 2012). Mino et al. (2013) use multi-locus sequence analysis to show that isolates of the chemosynthetic thermophilic genus *Persephonella* from different vent sites in the Okinawa Trough and in the distant Southern Mariana Trench in the western Pacific undergo sympatric speciation. Similarly, Wagner et al. (2013) use multi-locus sequence analysis to demonstrate genetic differentiation among strains of the thermophilic fermenting Firmicute, *Thermoanaerobacter uzonensis*, from different terrestrial hot spring locations within the Uzon Caldera in Kamtchatka. In this case, genetic divergence did not correlate with geographic separation on these short distances of mostly a few 100 m. Sylvan et al. (2013) describe how the biogeographical boundary of the north-south transition from basalt-hosted to andesite-hosted vents on the Eastern Lau spreading Center and Valu Fa ridge, appears to select for distinct bacterial taxa on silicate rocks, whereas inactive sulfides show major differences in bacterial community structure between the surface and the interior sulfide mineral matrix. Members of the Zetaproteobacteria, including the microaerobic and chemolithotrophic iron oxidizer *Mariprofundus ferrooxidans*, fall into different 16S rRNA phylotypes of Zetaproteobacteria from the Southern Mariana Arc, the Hawaiian hot spot, and from the Vai'lulu/Tonga Arc/East Lau Spreading Center/Kermadec Arc. This study shows that the Zetaproteobacteria from these sites form mutually intertwined microclusters on the 16S rRNA gene level, suggesting more detailed genomic analyses to explore their biogeography in fine resolution (Singer et al., 2013).

This research topic includes methodological assessments. Molecular surveys of biogeographic patterns and dominant microbial populations depend critically on the molecular tools used. To document inherent methodological biases, Hoshino and Inagaki (2013) perform a comparative molecular analysis of bacterial populations in hydrothermal sediments of Yonaguni Knoll in the Southern Okinawa Trough. By comparing the phylotypes recovered by conventional reverse-transcription PCR with domain-specific primers and by previous poly-A tailing of the extracted rRNA, they find different clades of Deltaproteobacteria and detect unusual archaea in the poly-A tailing assay that may escape detection by conventional PCR or RT-PCR using domain-specific primers.

The last two contributions to this research topic discuss microbial groups that are not commonly considered as components of hydrothermal microbiota, marine heterotrophic bacteria and pelagic marine archaea. When cosmopolitan, heterotrophic marine bacteria appear in molecular diversity surveys of hydrothermal vents or other extreme habitats, they are regarded as “hitchhikers” or potentially contaminants from entrainment of seawater. Handley and Lloyd (2013) examine the cosmopolitan marine genus *Marinobacter* and find that these versatile bacteria—commonly regarded as aerobes or fermenters—include nitrate-reducing and metal-reducing representatives from low-temperature vents, marine sediments and mid-ocean ridge basalts. This genus is therefore well-suited to colonize aerobic- to-anoxic gradients in most marine environments, including hydrothermal vents. Likewise, the Thaumarchaeota—marine archaea that are ubiquitous in the cold and oxic marine water column—have been found in surficial marine sediments, hydrothermal plumes, and hot spring sediments. Ragon et al. (2013) report a new phylotype within the terrestrial hot spring branch of the Thaumarchaeota, detected in deep hot (60°C) spring waters in Naica Mine, Chihuahua, Mexico. Thus, the Thaumarchaeota emerge as one of the most adaptable archaeal lineages that inhabit marine and terrestrial, oxic and anoxic, cold, and hydrothermal habitats.

These papers represent a sampler of ongoing microbiological research in hydrothermal environments, and demonstrate the wide multidisciplinary context of this field. Hydrothermal vents are not disconnected in time and space from the wider deep-sea ecosystem, without geological and biogeographical context. The research papers assembled here integrate geology, biogeochemistry, microbial physiology, microbial genomics and systematics across spatial scales that zoom in and out depending on the research question at hand. Perhaps no other field is so intertwined with the geological and geochemical evolution of the oceans, and promises so many biochemical and physiological discoveries still to be made within the unexhausted richness of extreme microbial life.

## Conflict of interest statement

The authors declare that the research was conducted in the absence of any commercial or financial relationships that could be construed as a potential conflict of interest.
